# Erector spinae plane block versus its combination with superficial parasternal intercostal plane block for postoperative pain after cardiac surgery: a prospective, randomized, double-blind study

**DOI:** 10.1186/s12871-022-01832-0

**Published:** 2022-09-16

**Authors:** Burhan Dost, Cengiz Kaya, Esra Turunc, Hilal Dokmeci, Semih Murat Yucel, Deniz Karakaya

**Affiliations:** 1 Department of Anesthesiology and Reanimation, Faculty of Medicine, Ondokuz Mayis University, Samsun, Turkey; 2 Department of Cardiovascular Surgery, Faculty of Medicine, Ondokuz Mayis University, Samsun, Turkey

**Keywords:** Postoperative pain, Acute, Surgical procedures, Cardiac, Median sternotomy, Nerve block, Ultrasonography

## Abstract

**Background:**

We aimed to compare the effectiveness of bilateral erector spinae plane (ESP) block and superficial parasternal intercostal plane (S-PIP) + ESP block in acute post-sternotomy pain following cardiac surgery.

**Methods:**

Forty-seven patients aged between 18 and 80 years of age with American Society of Anesthesiologists class II–III due to undergo median sternotomy for cardiac surgery were included in this prospective, randomized, double-blinded study. Following randomization into two groups, one group received bilateral ultrasound-guided ESP and the other S-PIP plus ESP block. Morphine consumption within the first 24 h after surgery was the primary outcome of the study while NRS scores at rest, NRS scores when coughing, time taken until extubation, use of rescue analgesic, presence of nausea/vomiting, length of hospital and intensive care unit (ICU) stay, and patient satisfaction were secondary outcome measures.

**Results:**

Morphine use up to 24 h following surgery was statistically significantly different between the ESP block and ESP + S-PIP block groups (18.63 ± 6.60 [15.84–21.41] mg/24 h vs 14.41 ± 5.38 [12.08–16.74] mg/24 h, *p* = 0.021). The ESP + S-PIP block group had considerably reduced pain scores compared to the ESP block group across all time points. Rescue analgesics were required in 21 (87.5%) patients in the ESP block group and seven (30.4%) in the ESP + S-PIP group (*p* < 0.001). PONV, length of stay in the ICU and hospital, and time to extubation were similar between groups.

**Conclusions:**

In open cardiac surgery, the combination of ESP and S-PIP blocks lowers pain scores and postoperative morphine requirement of patients.

**Trial registration:**

Clinicaltrials Registration No: NCT05191953, Registration Date: 14/01/2022.

**Supplementary Information:**

The online version contains supplementary material available at 10.1186/s12871-022-01832-0.

## Background

In cardiac surgery, it is well known that good postoperative pain management positively affects clinical outcomes [1]. Although the pain is usually related to the sternotomy, rib/intercostal nerve injury, parietal pleura, pericardium, and chest drain placement resulting from sternal retraction also contribute to postoperative pain. Recently, ultrasound (US)-guided interfacial plane blocks have been used as a part of multimodal analgesia to treat such pain [1, 2]. Forero et al. first described erector spinae plane (ESP) block as the administration of local anesthetic (LA) in to the interfacial plane between the erector spinae muscles and the transverse processes of the thoracic vertebrae [3]. Theoretically, it is thought that the LA spreads both craniocaudally and anteriorly, via the foramina costotransverse. Thus, the ventral/dorsal rami of spinal nerves, dorsal root ganglion, and rami communicants can be blocked at multiple levels [4, 5].

In patients receiving a sternotomy for cardiac surgery, bilateral thoracic ESP catheters, or the single-injection technique decreased the use of opioids and pain scores [6–8]. On the contrary, studies of LA distribution during ESP blockade have reported that, contrary to the predicted mechanism, the spread of anesthetic in the paravertebral region is quite inconsistent and unpredictable, and involvement is often limited to the dorsal ramus [9, 10]. For example, Taketa et al. found that the number of anesthetized dermatomes in the parasternal area was quite low, even with continuous LA infusion for up to 20 h with thoracic ESP [11]. Superficial parasternal intercostal block (S-PIP), a facial block that provides analgesia in the parasternal area, has been shown to reduce opioid consumption after median sternotomy by blocking the anterior cutaneous branches of the thoracic intercostal nerves (Th2-6) [12]. The S-PIP block may increase analgesic efficacy when combined with the ESP block, as distribution is inconsistent with the ESP block. Therefore, the hypothesis in our study was, adding S-PIP block to ESP block in cardiac surgery reduces postoperative morphine consumption. This study's aim was to measure the effectiveness of bilateral ESP block and S-PIP + ESP block when given as a component of multimodal analgesics in patients undergoing cardiac surgery with a median sternotomy. The primary outcome was the total morphine usage within 24 h after surgery.

## Methods

### Study design

This single-centered, prospective, randomized, double-blinded study was performed at the Ondokuz Mayis University from January to June 2022. The study was approved by the Clinical Research Ethics Committee of Ondokuz Mayis University (approval number: 2021/380) and informed consent was obtained from all subjects. The study was conducted according to the ethical principles of the Declaration of Helsinki and was registered on Clinicaltrials.gov (Registration No: NCT05191953 Registration Date: 14/01/2022) before the first patient enrollment. There were no changes made to the study protocol after trial commencement. In addition, the study was planned and conducted in accordance with the Uniform Guidelines for the Reporting of Studies (CONSORT) [13].

### Study population

Patients aged 18 to 80 years, who were due to undergo median sternotomy for the purpose of elective on-pump cardiac surgery, and who had an American Society of Anesthesiologists (ASA) physical status classification II–III were included. The exclusion criteria were patients: with cognitive impairment (patients unable to assess verbal numeric pain scales); undergoing emergency or reoperation, minimally invasive procedures; who refused participation in the study; and with preexisting hypersensitivity or allergy to LA, severe dysfunction of a major organ (e.g., renal or hepatic failure), left ventricular ejection fraction less than 30%, mental disorders, pregnant or lactating patients, hematologic disorders, alcohol/drug abuse, and/or daily opioid use.

### Randomization and blinding

Patients were allocated into one of the two groups using randomization, (1:1 ratio, parallel randomization) using the computer program "Research Randomizer" (https://www.randomizer.org/), which was run by a team member who was not involved in surgery or patient assessment. The same team members prepared opaque envelopes that concealed the type of intervention. An assistant who was not actively involved in the study had each patient choose a sealed envelope that contained a participation number. These envelopes were opened a few minutes before the regional block initiative. The researchers, patients, surgeons, and nurses were blinded to the randomization of the groups. To ensure quality and consistency of the blocks, a single anesthesiologist who had an experience of conducting both blocks at least 50 times administered each block. For blinding, this anesthesiologist not involved in intraoperative anesthetic management and data collection. For 24 h following surgery, a resident of anesthesia who was not involved in the procedures followed the patients and recorded data.

### Anesthesia management

Perioperative management was conducted in accordance with standard care protocols for cardiac anesthesia at our institute. Upon arrival to the operating room, standard ASA monitoring (electrocardiography, pulse oximetry, non-invasive blood pressure measurement, and bispectral index) was performed. An arterial line was inserted with a local anesthesia to monitor arterial pressure. Following tracheal intubation, for central venous pressure monitoring, a central venous catheter was inserted through the right internal jugular vein. Intravenous (IV) midazolam 0.05–0.1 mg/kg, fentanyl 2–5 µ/kg IV, pentothal 4–5 mg/kg IV), and rocuronium (1 mg/kg IV) was utilized for induction. Anesthesia was maintained using inhalation sevoflurane (MAC 1), O_2_/air (FIO_2_ 0.40), and IV fentanyl infusion (2–5 µ/kg/hour), targeting bispectral index values of 40–60, and hemodynamic parameters (heart rate and blood pressure) within 20% of baseline. All patients were given 0.05 mg/kg IV morphine at the end of the procedure before being transferred to the intensive care unit (ICU).

### Ultrasound guided bilateral ESP block

**Fig. 1:**
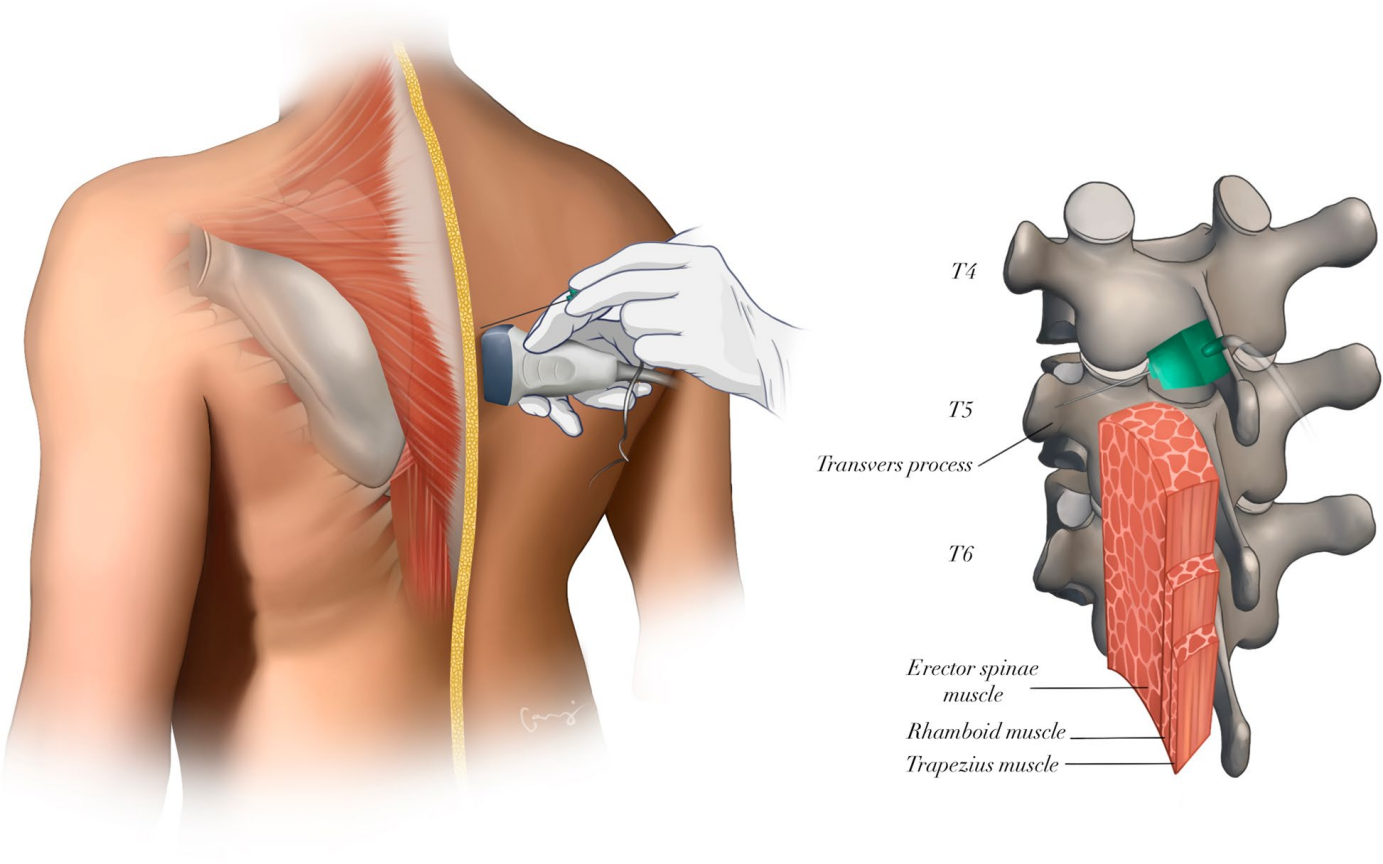
Schematic illustration of an ultrasound-guided erector spinae plane block

Before induction of general anesthesia, the ESP block was performed in the operation room as we previously described [14]. To keep Ramsay sedation scores at 2 (patient is awake, calm and watching their surroundings), infusions of midazolam (0.02 mg/kg) and remifentanil (0.05–0.1 µ/kg/min) were administered. After establishing aseptic conditions, the vertebral prominences (C7) were first palpated in the sitting position, and counting of the vertebral processes allowed for the precise localization of the T5 spinous process. The US probe (8–13 MHz, LOGIQ V1, GE Healthcare, USA) was initially placed in the middle vertebral line at the T5 level in the sagittal plane. The erector spinae muscle and transverse process were visualized by moving the US probe approximately 3–4 cm laterally from the midline. After the patient's skin was anesthetized by infiltration of 2 mL of 2% lidocaine, the block needle (21G 100 mm SonoPlex STIM Pajunk, Germany) was inserted using an in-plane approach until it touched the T5 transverse process. The needle tip was positioned in the fascial plane on the deep surface of the erector spinae muscle (Fig. 1). After confirmation of needle tip placement was made by hydrodissection of the interfascial plane with 2 mL of normal saline, 20 mL of 0.25% bupivacaine (Marcaine®, Astra Zeneca, US) was administered. During the injection, craniocaudal distribution of the LA could be seen in real time. On the opposing side, the procedure was carried out in exactly the same way.

### US-guided bilateral S-PIP block

**Fig. 2:**
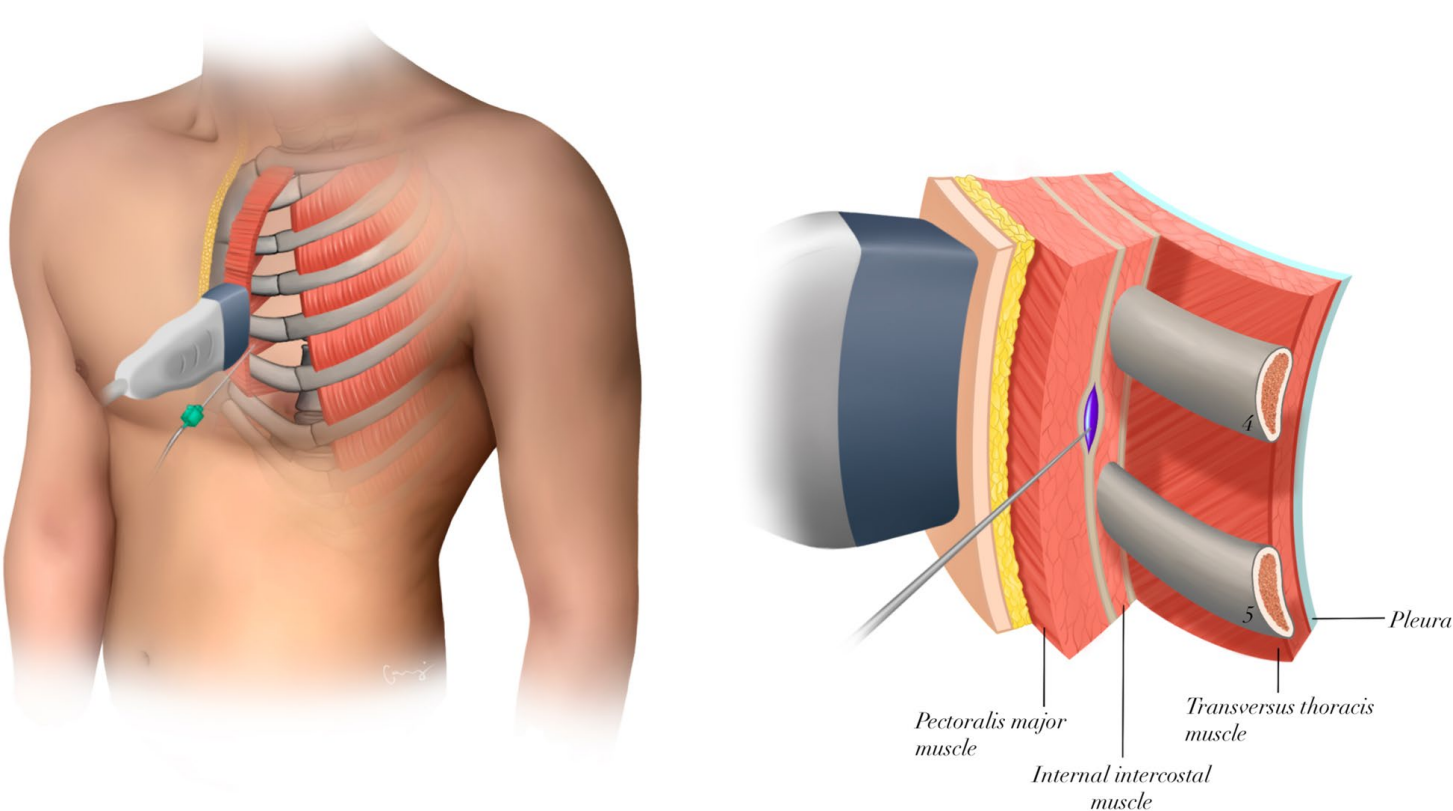
Schematic illustration of where to inject local anesthetic when using an ultrasound-guided superficial parasternal intercostal plane block. Purple highlighted area is the desired spread of local anesthetic

In the ESPB + S-PIP group, the S-PIP block [15] was administered under US guidance, in addition to the ESP block. After intubation and before the surgical procedure began, the injection site was prepped while the patients were in the supine position. A linear US probe was placed between the 4th and 5th intercostal spaces, 2–3 cm lateral of the parasagittal midline, and the depth of the ultrasound image was set to be around 2 to 3 cm. The block needle was then advanced in the caudocranial direction and injected LA into the fascial plane between pectoralis major, and internal intercostal muscles (Fig. 2). To confirm the location of the needle tip, hydrodissection with 1–3 mL 0.9% saline was performed to observe administration of the saline in to the interfascial area. Following application of negative pressure, 10 mL 0.25% bupivacaine was injected into the interfacial area. For a successful application, craniocaudal diffusion of the LA was simultaneously observed during the injection. The same steps were taken to complete the process on the other side. Care was taken that the maximal dose of 2.5 mg/kg (ideal body weight) bupivacaine was not exceeded.

### Postoperative analgesia

Herein, the first postoperative 24 h refers to the first postoperative 24 h that patients spent in the ICU after extubation. All patients received 1 g of IV acetaminophen every eight hours for the first 24 h as part of multimodal analgesia. At a dose of 20 µ/kg morphine, lockout time of 10 min, and the 4-h limit at 80% of the maximum possible dose, postoperative IV patient-controlled analgesia (PCA) (Body Guard 575 pain manager, UK) was provided for all patients. If numerical rating scales [NRS] score was ≥ 4 despite PCA, tramadol 100 mg IV (maximum 300 mg/day) was used for rescue analgesia, within 30 min. Two distinct methods were utilized for assessment of postoperative pain using NRS: at rest and during coughing at extubation, after three, six, 12, 18, and 24 h. The NRS is a scale where pain is scored from 0 to 10. A score of 0 is given for no pain and a score of 10 represents the worst possible pain that can be imagined. All patients were educated regarding NRS use of a PCA device, the day before the scheduled surgery. The patient's need for opioids from the PCA device was based on their NRS score at rest. Patients were advised to request opioids if their resting NRS score were above four.

To assess patient satisfaction with management of pain, the Turkish version of the Revised American Pain Society Patient Outcome Questionnaire (APS-POQ-R) [16] was utilized. The The APS-POQ-R is a pain management quality improvement tool for use in adult hospitals. It evaluates six different facets of care, including (1) pain severity and relief; (2) the impact of pain on activity, sleep, and negative emotions; (3) medication side effects; (4) the usefulness of pain treatment information; (5) the ability to participate in pain treatment decisions; and (6) the use of nonpharmacological techniques.

### Postoperative nausea and vomiting

We used a verbal, descriptive scale to rate the intensity of postoperative nausea and vomiting (PONV) at 0, 3, 6, 12, 18, and 24 h following extubation. When the score was less than three, an intravenous dose of four milligrams of ondansetron was given, with a second dose given eight hours later, if necessary. There was a four-point scale for PONV, with zero indicating no nausea, one indicating mild feeling of nausea, two indicating moderate feeling of nausea, three severe nausea, and four indicating repeated vomiting.

### Outcomes

The primary outcome of our study was the use of morphine within the first 24 h after surgery. NRS scores at rest, NRS scores during coughing, time to extubation, use of rescue analgesic, nausea/vomiting, length of stay in the ICU and hospital, and patient satisfaction were the secondary outcomes. Demographic data (age, sex, and body mass index [BMI]), type and duration of surgery, ASA scores, comorbidities, time under cardiopulmonary bypass, aortic cross-clamp, intraoperative total fentanyl consumption, opioid-related adverse events, and block-related complications were recorded in both groups.

Data from our pilot study of 10 patients per group revealed total PCA morphine use to be 21.1 ± 7.8 mg for the ESP block group and 13.9 ± 4.9 mg for the ESP + S-PIP block group. Calculations with G-Power (Heinrich-Heine-Universität Düsseldorf, Düsseldorf, Germany) using a type 1 error of 0.05 and power of 0.95, demonstrated that the minimum number of cases that must be present in each group in order to identify a statistically significant difference between the two means was 19. Given the possibility of data loss, the investigators opted to include 23 participants in each group, representing a 20% increase.

### Statistical analysis

Data analysis was conducted using IBM’s SPSS Software (version 23.0, IBM, New York, USA) Normal distribution was assessed using the Shapiro–Wilk test. The independent two-sample t-test was utilized to compare normally distributed data by paired groups, whilst the Mann–Whitney U test was used to compare non-normally distributed data. The Yates correction, Pearson’s chi-square test, and Fisher’s exact test were used to examine the relationship between categorical variables by the group. The Friedman test was used for non-normally distributed data to examine changes in the values over time. Dunn’s test with Bonferroni correction was used for multiple comparisons. The analysis results are presented as mean ± standard deviation (95% confidence interval [CI]) and median (interquartile range) for quantitative data and frequency (percent) for categorical variables. The significance level was set at *p* < 0.05.

## Results

**Fig. 3:**
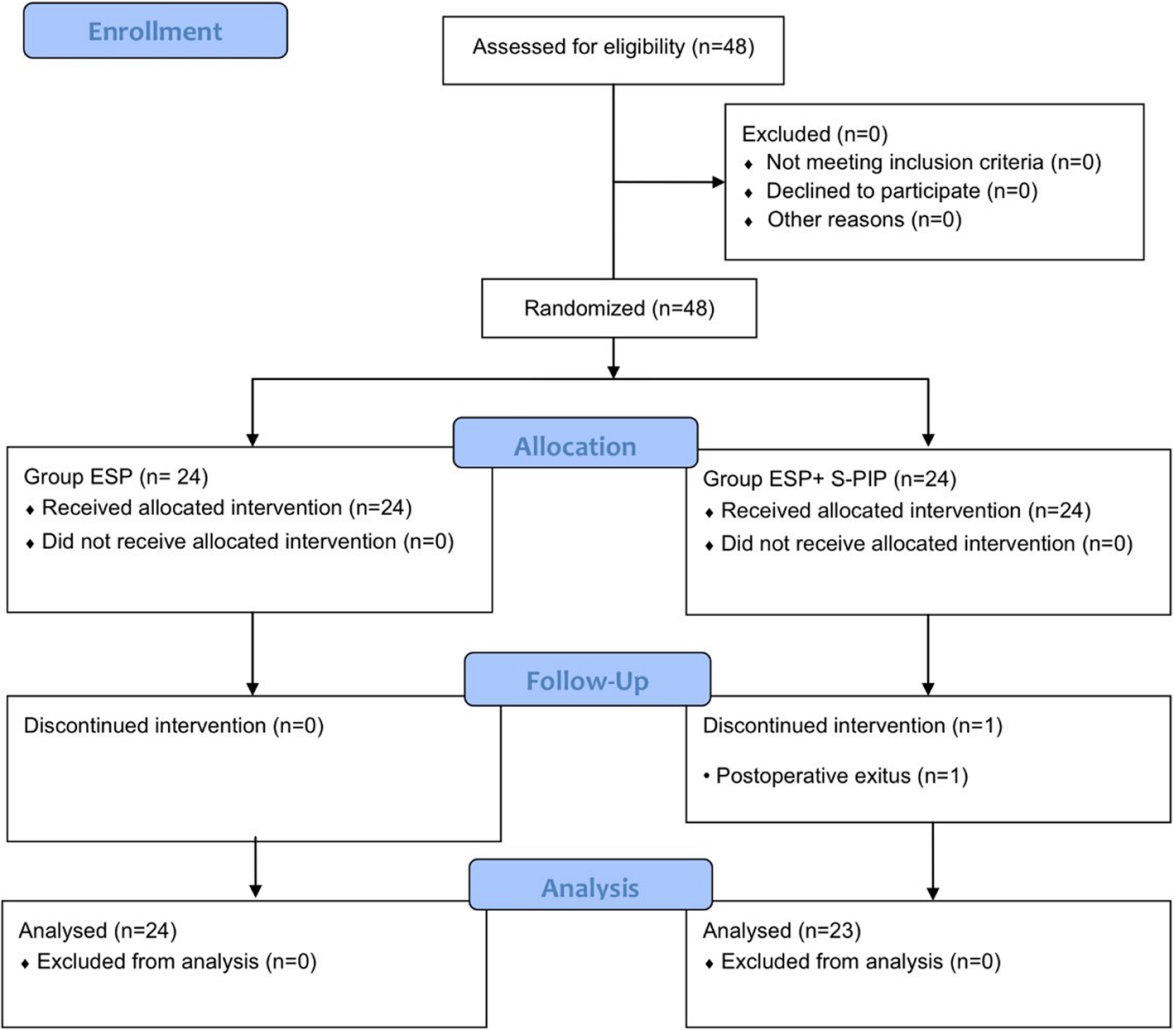
Flow diagram showing the distribution of patient data. Abbreviations: ESP, erector spinae plane; S-PIP, superficial parasternal intercostal plane

In total, 48 participants were assigned to either of two groups, through randomization. However, one patient in the ESP + S-PIP block group died during follow-up; thus, the final number of patients participating in the study was 47. A participant flow diagram is shown in Fig. 3. Patient demographics, comorbidities, and surgical characteristics are demonstrated in Table 1. There was no difference between the groups with regards to comorbidities, despite the presence of various comorbidities in both groups.

**Table 1:** Patient demographic and surgical characteristics and clinical outcomes

	**Group ESP (*n* = 24)**	**Group ESP + S-PIP (*n* = 23)**	***p* value**
Sex, female/male, n (%)	7 (30) / 17 (70)	9 (39.1) / 14 (60.9)	0.680
Age, years	58.13 ± 11.79 (53.15—63.1)	57.48 ± 10.96 (52.74—62.22)	0.847
BMI, kg/m^2^	27.86 ± 4.48 (25.97—29.75)	28.37 ± 5.18 (26.13—30.61)	0.719
ASA (II/ III), n (%)	5 (20.8) / 19 (79.2)	6 (26.1) / 17 (73.9)	0.671
Comorbidities, n (%)			
None	3 (12.5)	3 (13)	
Cardiovascular system^a^	9 (37.5)	5 (15.2)	
Endocrine system^b^	2 (8.3)	3 (13)	
Respiratory system^c^	0 (0)	1 (4.3)	0.650
> 1 more system	10 (41.7)	10 (43.5)	
Other	0 (0)	1 (4.3)	
Surgery type, n (%)			
CABG	15 (62.5)	12 (52.2)	
AVR	2 (8.3)	6 (26.1)	
MVR	2 (8.3)	1 (4.3)	
ASD	1 (4.2)	1 (4.3)	0.431
CABG + MVR	2 (8.3)	0 (0)	
CABG + AVR	2 (8.3)	1 (4.3)	
Atrial myxoma	0 (0)	1 (4.3)	
Ventricular thrombus	0 (0)	1 (4.3)	
Ejection fraction %	60 (46.5—60)	55 (50—60)	0.878
Sternal retraction distance (cm)	13 (12—13)	13 (13—14)	0.051
Duration of surgery (min)	270 (250—300)	255 (230—270)	0.071
Bypass time (min)	128.5 ± 35.1 (113.7—143.4)	108.22 ± 30.30 (95.12—121.32)	**0.039**
Cross-clamp time (min)	81.5 (56—102.5)	67 (56—80)	0.250
Extubation time (min)	225.9 ± 81.3 (191.6—260.3)	250.6 ± 67.6 (221.4—279.9)	0.265
ICU discharge time (h)	50 (25.5—98)	48 (24—72)	0.254
Hospital LOS time (h)	163 (145—240)	168 (144—192)	0.429
Intraoperative fentanyl consumption (μg/kg/min)	0.05 (0.04—0.06)	0.04 (0.03—0.05)	**0.045**
Morphine consumption first 24 h (mg)	18.63 ± 6.60 (15.84—21.41)	14.41 ± 5.38 (12.08—16.74)	**0.021**
Patients given rescue analgesic in first 24 h, n (%)	21 (87.5)	7 (30.4)	** < 0.001**

Continuous variables are presented as median (interquartile range) or mean ± standard deviation and categorical variables are presented as counts (percentages). Statistically significant difference is highlighted in bold

*Abbreviations:ASA* American Society of Anesthesiologists, *ASD *atrial septal defect, *AVR* aortic valve replacement, *BMI* body mass index, *CABG* coronary artery bypass grafting, *ESP* erector spinae plane, *ICU* intensive care unit, *LOS* length of stay time, *MVR* mitral valve replacement, *S-PIP* superficial parasternal intercostal plane

^a^Hypertension

^b^Type 2 diabetes, goiter

^c^Asthma

Morphine consumption in the first postoperative 24 h, which is the primary outcome of our study, showed a statistically significant difference between the ESP block and ESP + S-PIP block groups (18.63 ± 6.60 [95% CI: 15.84–21.41] mg/24 h vs. 14.41 ± 5.38 [95% CI: 12.08–16.74] mg/24 h, respectively, *p* = 0.021) as shown in Table 1.

**Fig. 4:**
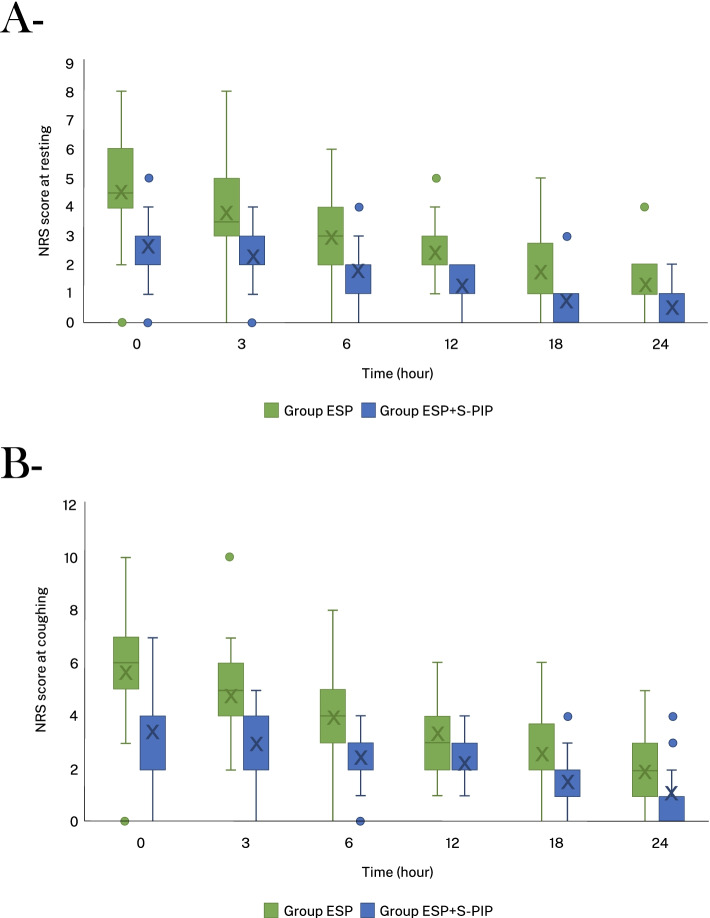
**A**, **B** Comparison of postoperative NRS_rest_ and NRS_coughing_ pain scores between the study groups at different time points. NRS values were significantly lower in ESP + S-PIP Block group than in ESP Block group at all time intervals postoperatively (*p* < 0.05). Abbreviations: NRS, numeric rating scale; ESP, erector spinae plane; S-PIP, superficial parasternal intercostal plane

There were also significant differences in NRS values between the ESP block and ESP + S-PIP block groups during rest and coughing. The ESP + S-PIP block group had considerably lower pain scores than the ESP block group across all time points (*p* < 0.05) (Fig. 4 and Supp. 1). NRS scores both at rest and when coughing were examined using a linear mixed-effects model, which revealed that there was a significant interaction between treatments and time (*p* < 0.001). In our study, the number of patients who received rescue analgesics was 21 (87.5%) in the ESP block group and seven (30.4%) in the ESP + S-PIP group (*p* < 0.001) (Table 1). The incidence of PONV, length of ICU stay, time of extubation, and length of hospital stay were similar in the two groups (Table 1 and Supp. 2). Neither group observed opioid-related adverse events (itching, bladder globe etc.) or block-related (hematoma formation, pneumo/hemothorax, infection or systemic LA toxicity etc.) were complications.

As detailed in Table 2, the patients in the ESP + S-PIP block group performed better with regards to the pain intensity domain of the APS-POQ-R questionnaire during the initial 24 h after surgery [most severe pain, median (interquartile range [IQR]), 5 (4–6) vs. 6 (5–7), *p* < 0.001, respectively]. Those in the ESP block group experienced a greater percentage of severely painful episodes in the first 24 h when compared to the ESP + S-PIP block group [median (IQR) 60 (50–65) vs. 50 (40–60), *p* < 0.001, respectively]. The percentage of pain relief in the first 24 h was higher in the ESP + S-PIP block group than in the ESP block group [median (IQR) 100 (70–100) vs. 70 (50–80), *p* < 0.001, respectively]. Impairment or hindrance of activities and sleep status due to pain was lower in the ESP + S-PIP block group (*p* < 0.05). Moreover, patient satisfaction was higher in the ESP + S-PIP block group when compare to the ESP block group [median (IQR) 10 (10–10), 9.5 (9–10), *p* < 0.001, respectively]. When comparing adverse effects, the groups did not differ (*p* > 0.05) (Table 2).

**Table 2:** APS‐POQ‐R questionnaire results 24 h after surgery

	**Group ESP** (*n* = 24)	**Group ESP + S-PIP** (*n* = 23)	***p* value**
**Pain intensity**			
Least pain	1 (0—2)	0 (0—1)	0.003
Worst pain	6 (5—7)	5 (4—6)	< 0.001
% of time in severe pain in the first 24 h	60 (50—65)	50 (40—60)	< 0.001
% of pain relief in the first 24 h	70 (50—80)	100 (70—100)	< 0.001
**Pain interfered or prevented activites**			
In the bed	4.5 (2.5—7)	2 (0—5)	0.004
Out of bed	5 (3.5—7.5)	2 (0—5)	0.003
**Pain interfered or prevented sleep**			
Falling asleep	3 (0.5—5.5)	0 (0—1)	0.001
Staying asleep	2 (0—5)	0 (0—1)	0.005
**Pain caused you to feel**			
Anxious	0.5 (0—3)	0 (0—0)	0.012
Depressed	0 (0 – 2)	0 (0 – 0)	0.009
Frightened	0 (0 – 0)	0 (0 – 0)	0.084
Helplessness	0 (0 – 0)	0 (0 – 0)	0.084
**Side effect**			
Nause	0 (0—2)	0 (0—3)	0.963
Drowsiness	0 (0—0)	0 (0—0)	0.328
Itching	0 (0—0)	0 (0—0)	0.328
Dizziness	0 (0—0)	0 (0 – 0)	0.388
**Patient perception of their pain management**			
Participation in pain management	10 (10—10)	10 (10—10)	0.114
Satisfaction with pain management	9.5 (9—10)	10 (10—10)	0.001
**Use of nonpharmacological interventions, n (%)**			
0	23 (95.8)	16 (69.6)	–––
1	1 (4.2)	6 (26.1)	
2	0 (0)	1(4.3)	
**Encourage you to use, n (%)**			
Never	2 (8.3)	1 ( 4.3)	0.709
Sometimes	10 (41.7)	8 (34.8)	
Often	12 ( 50)	14 (60.9)	
**Information of patients’ pain treatment option, n (%)**			
Yes/no	24 (100) / 0 (0)	22 (95.7) / 1 (4.3)	0.302
**How helpful**	10 (9—10)	10 (10–10)	0.001
**Received help in completing the questionnaire, n (%)**			
Yes / No	24 (100) / 0 (0)	23 (100) / 0 (0)	**–––**

Data are presented as median (interquartile range). Statistics are presented for relevant patients responses to select questions from the Revised American Pain Society Outcome Questionnaire-TR Version

*Abbreviations:* *APS‐POQ‐R* American Pain Society Outcome Questionnaire- Revised, *ESP* erector spinae plane, *S-PIP* superficial parasternal intercostal plane

## Discussion

Our study has shown that the addition of the S-PIP block to the ESP block in cardiac surgery results in a reduction in 24 h postoperative morphine consumption, pain scores, and the number of patients receiving rescue analgesics. We observed no differences between the study groups in regards to the incidence of PONV, length of ICU stay, extubation time, or length of hospital stay.

Studies have shown that ESP block decreases postoperative morphine use in cardiac surgery [8, 17–19]. A recent meta-analysis has shown that parasternal block could relieve pain and limit opioid-related complications by reducing opioid consumption [20]. In our study, using S-PIP block and ESP block reduced morphine consumption (18.63 ± 6.60 mg vs. 14.41 ± 5.38 mg) by approximately 5 mg. According to reviewed literature, a decrease of 10 mg IV morphine within 24 h is the minimal clinically significant difference (MCID) for several operations [21]. However, the MCID value may not be the same for all types of surgeries and patients [22]. Although a statistically significant difference was found in our study, it is difficult to determine the magnitude of the analgesic effect of adding a superficial parasternal fascial block in this population since the decrease in morphine consumption that yields the MCID value has not yet been determined for a cardiac surgery procedure. The combination of ESP and S-PIP block appears to be a useful technique for facilitating recovery and improving patient satisfaction following open heart surgery, as evidenced by the reduction in pain scores, the number of patients who required rescue analgesics, as well as the satisfaction outcomes in the first postoperative 24 h.

The ESP block's precise mechanism(s) of action are still unknown, although it is has been suggested that the spread of the LA to the thoracic paravertebral space and associated neural structures is responsible [23]. However, some studies have obtained variable results and low failure rates. This may be due to the spread of the LA and numerous anatomic differences. It is not clear whether the LA spreads in the paravertebral or epidural areas in the vertical plane or whether the cutaneous side branches are blocked in the interfascial plane, and even if the LA reaches all these areas, or whether it encircles the nerve or the nerve root. Additionally, anatomic adhesions in the interfascial area may also lead to failure of the block in the applied area [24]. Although the ESP block has been found to effectively relieve pain after cardiac surgery, in the dermatome study of Taketa et al., the ESP block was shown to produce weaker dermatomal spread along the anterior skin branch compared with the lateral skin branch region [25].

S-PIP and deep parasternal intercostal block (D-PIP) are two newer truncal interfascial plane blocks used in cardiac surgery [26, 27]. In our previous study, which compared S-PIP with D-PIP for the management of post-sternotomy pain management after cardiac surgery, we found that they had similar effects on morphine consumption in the postoperative 24 h and as S-PIP is simpler to administer with a low risk of significant complications, we concluded that S-PIP might be a more attractive option for acute post-sternotomy pain management [15]. For this reason, we considered adding the S-PIP block to the ESP block in our study. We aimed to achieve better analgesic management by achieving a complete blockade of the chest wall’s anterior branches, which are not thought to be completely blocked during ESP blockade. In addition, Bousquet et al. showed in their study that each group involving ten cardiac surgery patients showed that the combination of bilateral parasternal and bilateral ESP blocks resulted in a significant reduction in morphine consumption compared to the control group. Looking at the data from this study, we observed that morphine consumption in the ESP + S-PIP group was similar to that in our study (14 mg vs. 12 mg) [28]. In addition, even if the combination of these two blocks produced a less marked reduction in the consumption of morphine, the fact that it is not excessively time-consuming or resource-consuming compared to a single block may justify us to choose it in clinical practice. However, it should be remembered that we used it in addition to bilateral ESP block, and bilaterally applied S-PIP block also carries some risks. Complications [29] such as hematoma, infection, and LA systemic toxicity, which may result from these blocks and loss of time, should also be considered.

In this research, we utilized the widely used APS-POQ-R as a meaningful measure of postoperative pain management quality. After open cardiac surgery, we found that combining ESP with S-PIP block was more beneficial than either modality alone in reducing postoperative pain intensity and enhancing sleep quality. In addition, the ESP + S-PIP block group reported greater overall satisfaction with pain management than the ESP block group.

This study has some limitations. First, the dermatomal analysis could not be performed in both groups because the S-PIP block was performed under general anesthesia, as in our routine practice. Second, the single-center structure of the study and the small number of patients may also be considered a limitation. Third of all, there was no no-block or sham-block control group in the trial. Although this is a drawback, this strategy was chosen since the available data suggests that S-PIP and D-PIP blocks are both useful for treating post-sternotomy pain. Nevertheless, the technique is still considered to be restrictive. Last of all, for consistency, blocks in this study were conducted by a single anesthesiologist; however, this also reduced external validation. It would be more appropriate to have blocks performed by more than one person to reduce physician-related reasons.

## Conclusions

In this study, the combination of ESP and S-PIP blocks modestly reduced postoperative morphine use and pain scores in patients undergoing open cardiac surgery.

## Supplementary Information

**Additional file 1.**

**Additional file 2.** 

## Data Availability

The datasets used and/or analysed during the current study available from the corresponding author on reasonable request.

## Abbreviations

US: Ultrasound
ESP: Erector Spinae Plane
LA: Local Anesthetic
S-PIP: Superficial Parasternal Intercostal Block
ASA: American Society of Anesthesiologists
ICU: Intensive Care Unit
PCA: Patient-Controlled Analgesia
NRS: Numerical Rating Scales
APS-POQ-R: Revised American Pain Society Patient Outcome Questionnaire
PONV: Postoperative Nausea and Vomiting
BMI: Body Mass Index
CI: Confidence Interval
MCID: Minimal Clinically Important Difference
