# Supplementary material for: Erector spinae plane block versus its combination with superficial parasternal intercostal plane block for postoperative pain after cardiac surgery: a prospective, randomized, double-blind study
Source: BMC Anesthesiol. 2022 Sep 16;22:295. doi: 10.1186/s12871-022-01832-0 (PMC9479438; doi:10.1186/s12871-022-01832-0)
Supplement: Supplementary file 1 — Additional file 1. [file 12871_2022_1832_MOESM1_ESM.docx]

**Supp.1.** Comparison of postoperative NRS pain scores between the study groups at different time points

|  | **Group ESP**  **(n=24)** | **Group ESP + S-PIP**  **(n=23)** | **p value** |
| --- | --- | --- | --- |
| **NRSrest** |  |  |  |
| 0^th^ hour | 4.5 (4 - 6) | 3 (2 - 3) | <0.001 |
| 3^rd^ hour | 3.5 (3 - 5) | 2 (2 - 3) | <0.001 |
| 6^th^ hour | 3 (2 - 4) | 2 (1 - 2) | 0.006 |
| 12^th^ hour | 2 (2 - 3) | 1 (1 - 2) | <0.001 |
| 18^th^ hour | 1 (1 - 2.5) | 1 (0 - 1) | 0.003 |
| 24^th^ hour | 1 (1 - 2 | 0 (0 - 1) | 0.002 |
| **NRScoughing** |  |  |  |
| 0^th^ hour | 6 (5 - 7) | 4 (2 - 4) | <0.001 |
| 3^rd^ hour | 5 (4 - 6) | 3 (2 - 4) | <0.001 |
| 6^th^ hour | 4 (3 - 5) | 2 (2 - 3) | 0.001 |
| 12^th^ hour | 3 (2 - 4) | 2 (2 - 3) | 0.007 |
| 18^th^ hour | 2 (2 - 3.5) | 1 (1 - 2) | 0.004 |
| 24^th^ hour | 2 (1 - 3) | 1 (0 - 1) | 0.042 |

Data are presented as median (interquartile range).

**Abbreviations:** NRS, numeric rating scale; ESP, erector spinae plane; S-PIP, superficial parasternal intercostal plane.
