# Supplementary material for: Erector spinae plane block versus its combination with superficial parasternal intercostal plane block for postoperative pain after cardiac surgery: a prospective, randomized, double-blind study
Source: BMC Anesthesiol. 2022 Sep 16;22:295. doi: 10.1186/s12871-022-01832-0 (PMC9479438; doi:10.1186/s12871-022-01832-0)
Supplement: Supplementary file 2 — Additional file 2. [file 12871_2022_1832_MOESM2_ESM.docx]

**Supp.2.** Comparison of postoperative nausea and vomiting scores between the two groups

|  | **Group ESP**  (n=24) | **Group ESP + S-PIP**  (n=23) | **p value** |
| --- | --- | --- | --- |
| Extubation  0  1  2  3 | \| 16 (66.7) \| \| --- \| \| 2 (8.3) \| \| 3 (12.5) \| \| 3 (12.5) \| | \| 16 (69.6) \| \| --- \| \| 1 (4.3) \| \| 0 (0) \| \| 6 (26.1) \| | 0.229 |
| 3^rd^ h  0  1  2  3 | \| 18 (75) \| \| --- \| \| 3 (12.5) \| \| 1 (4.2) \| \| 2 (8.3) \| | \| 16 (69.6) \| \| --- \| \| 3 (13) \| \| 3 (13) \| \| 1 (4.3) \| | 0.698 |
| 6^th^ h  0  1  2 | \| 22 (91.7) \| \| --- \| \| 1 (4.2) \| \| 1 (4.2) \| | \| 22 (95.7) \| \| --- \| \| 1 (4.3) \| \| 0 (0) \| | **-** |
| 12^th^ h  0  1 | \| 23 (95.8) \| \| --- \| \| 1 (4.2) \| | \| 23 (100) \| \| --- \| \| 0 (0) \| | **-** |
| 18^th^ h  0 | 24 (100) | 23 (100) | **-** |
| 24^th^ h  0 | 24 (100) | 23 (100) | **-** |

Data are presented as counts (percentages). Postoperative nausea and vomiting score: 0 = none; 1 = mild nausea; 2 = moderate nausea; 3 = vomiting once; and 4 = vomiting more than once.

**Abbreviations:** ESP, erector spinae plane block; S-PIP, superficial parasternal intercostal plane.
